# Review of "In the Eye of the Needle: Diary of a Medically Supervised Injecting Centre" by Ingrid van Beek Allen & Unwin 2004

**DOI:** 10.1186/1477-7517-2-15

**Published:** 2005-09-15

**Authors:** Allan Clear

**Affiliations:** 1 Executive Director, Harm Reduction Coalition, 22 West 27th Street, 5th Fl, New York, NY 10001, USA

## 

However they are labeled, there are a couple of dozen safe injection facilities, safe injection rooms, safe injection spaces, drug consumption rooms, or medically supervised injecting centres around the globe. The most recent have appeared in Vancouver, Canada, and the most scrutinized is in Sydney, Australia. Dr. Ingrid Van Beek has diarized the early history of Sydney's centre in her book, "In the Eye of the Needle". This book is much more than the story of the medically supervised injection center. The lessons imparted here are invaluable for everyone working with drug users and have universal application. Van Beek skillfully weaves several different strands throughout the book including the mechanics of opening and running the center; the emotional toll it takes on staff; the humanity of the drug users the center serves; the need of the clients; and the scrutiny that an institution comes under for being deemed "controversial". Ultimately this book is about how compassion, healthcare, dignity and human rights can be obtained for drug users.

Medically supervised injection centres are controlled environments aimed at reducing the negative consequences of injecting in public places and are usually staffed by medical professionals and social workers. Injecting in uncontrolled spaces leads to missed and hurried shots, overdose risk and "offending" the general public. Supervised injection centres emerged in Western Europe in the 1990s, principally in Switzerland and Germany, and evaluations of the centres have shown them to be an effective intervention and a practical tool for health promotion among drug users. The centres are particularly appropriate in locations with thriving street based drug markets. As experienced in Vancouver and Sydney, it is not uncommon for injection rooms to be foreshadowed by activists who first set up illegal spaces and then gain legal status as a result of government support.

Despite the corny title, "In the Eye of the Needle" is the best book yet written on the experience of working in the field of harm reduction. It sounds like I'm damning with faint praise because, as far as I know, there are no other books that explore the worker's experience. But no, this is really a great book. You do not have to open a safe injection room to relate to everything that occurs herein. Involved in drug services, needle exchange, housing, mental health, drug treatment? The book covers, all too familiarly, the issues – site location, back stabbing by colleagues, under appreciation of hard working staff, tears, sweat and blood everywhere. More blood than you can possibly imagine. Blood on the walls and blood on floor. We're talking about people shooting up. Favorite drug users die and colleagues who you don't know are using, overdose. Opposing politicians and the media are scurrilous, self-serving, immoral hypocrites. At the same time, politicians and media come through with the support when needed, always by a hair's breadth and often without getting any substance behind the facts. It's a lonely business sometimes. The only people who seem to know what goes on are the workers. And the drug users. Read this book and plot out a media strategy.

Dr. Ingrid van Beek was already running primary health care services for drug users when she was contacted by a local police chief concerned about the volume of emergency calls for overdose situations. She took on the oversight of the medically supervised injecting centre on top of her day job and spent the next couple of years running from centre to centre. The organizing work was intense and all encompassing. Van Beek worked with everyone from the local residents and business groups to government representatives and law enforcement. At the same time, the United Nations International Narcotics Control Board decided to criticize the centre, overlooking for arcane reasons the pre-existing European injection spaces, causing more political fall out for the centre.

As a clinician, Dr. van Beek is the perfect foil for taking on controversial services. She handles the burdensome site visits from the police and health authorities with a stellar resignation. "Although I pointed out that no other health facility in the land is routinely inspected by licensing authorities without notice, it was argued that some in the community might expect this and we must be seen to be absolutely squeaky clean in all respects." Who in the harm reduction field does not feel that we are held to a different standard than other services? The medically supervised injecting centre, as described in the book, is clinical in design as opposed to the more relaxed community oriented European model of injection rooms. Smoking is not allowed. Users cannot hang out. In 18-months, the centre handled 554 overdoses without loss of life and thousands of injection episodes. The most poignant moment in the book is the frustration of receiving an evaluation that minimized the success of the centre by giving it a marginal passing grade. Rigor is fine in research but researcher rigor mortis is sad. The number of deaths the opening of the centre prevented cannot be quantified. Prevention is hard to prove conclusively. Too often research is cautious and verges on the ridiculously conservative. It seems this was the case here.

Aside from describing the creation of the centre, this book is a much-needed primer for overdose prevention. Overdose deaths can be largely prevented with appropriate and timely care. When the centre first opened, Sydney experienced a heroin drought and most users in distress were revived with oxygen. Only as the drought was ending and heroin purity increasing did the centre use naloxone, and then only infrequently. "Increasingly," van Beek writes, "I appreciate that the injecting centre provides a unique setting in which health care workers actually see the overdose occurring from the very outset, identifying symptoms of heroin (or whatever drug) overdose and administering appropriate treatment very soon thereafter. This can't and doesn't occur in any other circumstance. By treating an overdose so early in its course, the damage already done and its natural progression is reversed so that Narcan (used to start breathing) will no longer be needed in most cases. This is how injecting centres potentially reduce the morbidity (damage to vital organs, especially the brain) and the mortality otherwise associated with overdose in unattended situations, even when there is a very prompt and efficient ambulance service on hand."

Globally, overdose has not had the attention that HIV prevention for drug users has received. It is an issue, however, that deserves equal attention. Whereas syringe exchange programs emerged rapidly as an HIV prevention strategy in the United States at the beginning of the nineties – seven programs in the late 1980s to 90 programs five years later – overdose prevention and education, however, have not expanded in the same fashion. 5 campaigns in five cities or states in five years is inadequate considering, west of the Rockies, drug users are more at risk of dying of an opiate overdose than from HIV. In France, scaling up of buprenorphine (Subutex) has greatly reduced mortality from opiate overdose. In Russia overdose is common, syringe exchanges are stagnant, and substitution therapy is illegal. In such a milieu, it is hard to envision a supervised injection center. Van Beek's detailed account of such a center can help the uninformed understand the mechanics of overdose first hand. One of the strengths of the book is that it is detailed enough to be of universal help in guiding one to develop overdose interventions for drug users without having to open a centre.

Aside from overdose prevention, safe injection rooms are primarily thought of as nuisance abatement strategies. Although not touched upon in the book, they are good venues for safe injection education on reducing soft tissue infections and disease prevention. At the recent International Harm Reduction Conference in Belfast, staff from the Sydney medically supervised injecting centre talked about the lack of blood awareness and the poor injection techniques of injectors, despite years of education by providers. Investigation by staff at the centre revealed that education such as rotating veins or releasing tourniquets before injection weren't necessarily practical for the user. This poses new challenges for providers and users alike, but it is a path we need to travel together.

It has been less of a challenge to incorporate harm reduction as a national approach to drugs in countries with health care systems that help people when they get sick, as opposed to systems that benefit insurance and pharmaceutical companies when a consumer needs services. It is also less challenging in countries that recognize that drug users are citizens with rights. This book, however, points up that even within a country, such as Australia, which prides itself on pragmatism, it is still difficult to implement an intervention that can benefit the lives of drug users. The story of the development of the medically supervised injection centre is a classic case of harm reduction struggling to assert its worth. Despite the success of the centre, there is no intention of replicating the facility in other places in Australia. It has not inspired other countries to follow suit (except for Vancouver, Canada) and injection centres are taking the path of other interventions for drug users in that they will be adopted gradually at a glacial pace.

I would love to see a spate of books inspired by "In the Eye of the Needle: Diary of a Medically Supervised Injecting Centre". We could come up with our own "Catch 22" or "One Flew Over the Cuckoo's Nest" to match the Kafkaesque world we inhabit. In the meantime we have with "In the Eye of the Needle", a readable, intelligent, quality account of the creation of a harm reduction program.

